# Can heart rate sequences from wearable devices predict day-long mental states in higher education students: a signal processing and machine learning case study at a UK university

**DOI:** 10.1186/s40708-024-00243-w

**Published:** 2024-12-05

**Authors:** Tianhua Chen

**Affiliations:** School of Computing and Engineering, University of Huddersfield, Huddersfield, WYK HD1 3DH UK

**Keywords:** Student Wellbeing, Mental Health, Stress, Heart Rate, Wearable, Signal Processing

## Abstract

The mental health of students in higher education has been a growing concern, with increasing evidence pointing to heightened risks of developing mental health condition. This research aims to explore whether day-long heart rate sequences, collected continuously through Apple Watch in an open environment without restrictions on daily routines, can effectively indicate mental states, particularly stress for university students. While heart rate (HR) is commonly used to monitor physical activity or responses to isolated stimuli in a controlled setting, such as stress-inducing tests, this study addresses the gap by analyzing heart rate fluctuations throughout a day, examining their potential to gauge overall stress levels in a more comprehensive and real-world context. The data for this research was collected at a public university in the UK. Using signal processing, both original heart rate sequences and their representations, via Fourier transformation and wavelet analysis, have been modeled using advanced machine learning algorithms. Having achieving statistically significant results over the baseline, this provides a understanding of how heart rate sequences alone may be used to characterize mental states through signal processing and machine learning, with the system poised for further testing as the ongoing data collection continues.

## Introduction

The mental health of students in higher education has become an increasingly pressing issue, with growing evidence highlighting the heightened risk of stress, anxiety, and depression among this population [1, 2]. Research briefings from the UK Parliament indicate a sharp rise in the number of domestic students disclosing mental health conditions to their universities. Since 2010, this number has grown steadily, exceeding 5% during the 2020/21 academic year [3]. However, the reality may be even more concerning, as confidential surveys often report higher rates of mental health challenges. A survey by the mental health charity Student Minds in 2022 found that over half of students surveyed said they have a current mental health issue, while one quarter of students surveyed said having a current, diagnosed mental health issue [4]. These findings underscore the urgent need for enhanced mental health support and resources within higher education to address this growing issue.

The increasing prevalence of wearable devices like smartwatches and fitness trackers, known for their user-friendly design and effortless integration into daily routines, has made them more accessible than ever. These devices enable continuous, real-time data collection in a non-intrusive way, making them particularly valuable for monitoring and identifying early signs of mental health issues in students. At the same time, research is expanding on how the data gathered from these wearables can be leveraged to track and manage mental health within the university student population [5, 6].

For example, a study [7] explored the potential of heart rate variability (HRV) data from wearable biosensors, using deep neural networks to predict health indicators such as stress, anxiety, depression, and overall health. Another study [8] collected continuous biometric data and weekly survey responses to explore the link between sleep metrics and perceived stress. The analysis revealed significant associations between stress levels and several sleep-related measures and heart rate variability and respiratory rate. The study [9] focused on stress detection by analyzing physiological signals like electrodermal activity, skin temperature, and heart rates, alongside self-reported anxiety and stress levels, in a semi-controlled lab environment. In another semi-controlled settings [10], the HRV of university students was monitored during different stages of an exam to assess stress, with findings suggest HRV was highest post-exam, indicating stress relief, while it was lower before and during the exam. Another study [11] presents a broader review of how HRV correlates with various conditions such as stress, and exercise, utilizing signal processing and machine learning techniques. Their findings highlight that generally, reduced HRV is associated with higher levels of morbidity and stress, whereas higher HRV often signals good health. However, they also note that HRV detection accuracy during activities involving movement—such as exercise, video gaming, and driving-remains notably limited.

Measuring the variation in time intervals between consecutive heartbeats, HRV is increasingly used in research as a key indicator for tracking changes in mental health [7, 9–13]. This is due to that HRV provides a detailed analysis of how these intervals vary, reflecting the balance of the autonomic nervous system and offering insights into physiological responses to stress and relaxation, making it a favored choice for investigating mental states. However, obtaining accurate HRV measurements typically requires controlled conditions and the analytical approach and steps applied to obtain HRV measures can be seen as complex [14]. On the other hand, heart rate (HR) is simpler to measure and is broadly understood, making it ideal for monitoring physical activity and basic cardiovascular health. Although HR is less sensitive to psychological changes than HRV, its straightforward measurement and common presence in wearables makes it a potentially useful metric for gauging emotional states. This is particularly true when variations in heart rate patterns over time are carefully analyzed, although its effectiveness in tracking mental states is less explored in research compared to HRV.

Recent research on directly utilizing heart rate as a predictor of mental states is limited, with only a few studies tangentially addressing this connection, or often in a controlled setting for the measurement of its change in response to a designated stimuli. For instance, 21 physiological features including heart rates and their subsets were empirically evaluated in [15] for their effectiveness in predicting stress and anxiety states. However, the specific role of heart rate as a predictor remains unexplored. In a controlled setting, a randomized trial [16] assessed the impact of progressive muscle relaxation and music therapy on reducing pre-exam stress and improving academic performance in nursing students. The experimental group, which received the intervention, showed reduced heart rate, while the control group experienced increased heart rate, blood pressure, and cortisol levels. Another study [17] also sought to determine how baseline stress levels might influence changes in heart rates and heart rate variability, where the Trier Stress Test was employed to induce stress, during which resting and stress-phase ECGs were recorded, alongside inter-second heart rate measurements. While it revealed novel changes in these parameters between resting and stress states, the study was unable to uncover consistent patterns in HRV and heart rate changes during stress.

Indeed, while the studies mentioned above illustrate the utility of heart rate measurements in specific contexts or activities, there remains a gap in research examining the effectiveness of heart rate as a continuous indicator for overall day-long mental states, in a fully open environment. Existing research primarily focuses on heart rate responses to isolated stimuli or conditions, such as emotional triggers, stress-inducing tests, or sleep patterns. This gap suggests an opportunity for more comprehensive studies that would track heart rate continuously over the course of a day to analyze how it fluctuates across a sequence of different activities, each potentially inducing unique heart rate responses. Such research could explore whether fluctuations in heart rate, when considered in the context of daily routines, could provide a reliable gauge of overall mental state such as overall stress levels throughout the day.

The goal of this research is to explore whether continuous, day-long heart rate data collected by university students from a UK university using wearable devices in a natural, unrestricted setting can reliably indicate mental states, especially stress. The methodology employs advanced machine learning algorithms to model the complexities of day-long heart rate sequences. It is further enhanced by signal processing techniques that analyze both the frequency and time domains of the heart rate data, providing a comprehensive approach to understanding heart rate variations as potential indicators of mental states.

Section 2 outlines the data collection an processing, including data cleaning, interpolation, and resampling. Section 3 details the methods used to transform temporal heart rate data into the frequency domain using Fourier Transform and wavelet analysis, followed by modelling through advanced machine learning algorithms. The experimental results are presented in Sect. 4, with the study’s limitations and potential avenues for future research discussed in Sect. 5.

## Data

### Background

The data for this study were gathered at a public university in Northern England, UK, with the project receiving the required ethical approval from the university’s ethics committee. Participants utilized an in-house built IOS app to log their daily emotional states and submitted their general health and activity data as collected through the Apple HealthKit app via their Apple Watches. This research specifically focuses on assessing day-long stress, which participants self-reported in response to the question, “Have you had a stressful day?” with possible responses being positive or negative. Initially scheduled to begin earlier, data collection faced delays due to technical issues and eventually the actual data collection period spanned between December 4, 2023, and July 31, 2024. Although over a hundred students showed interest in participating earlier on, only six undergraduates could enroll, due to various reasons including the lack of necessary equipment. The subsequent sections detail the data collected and the steps involved in its processing.

### Data exploration

**Fig. 1:**
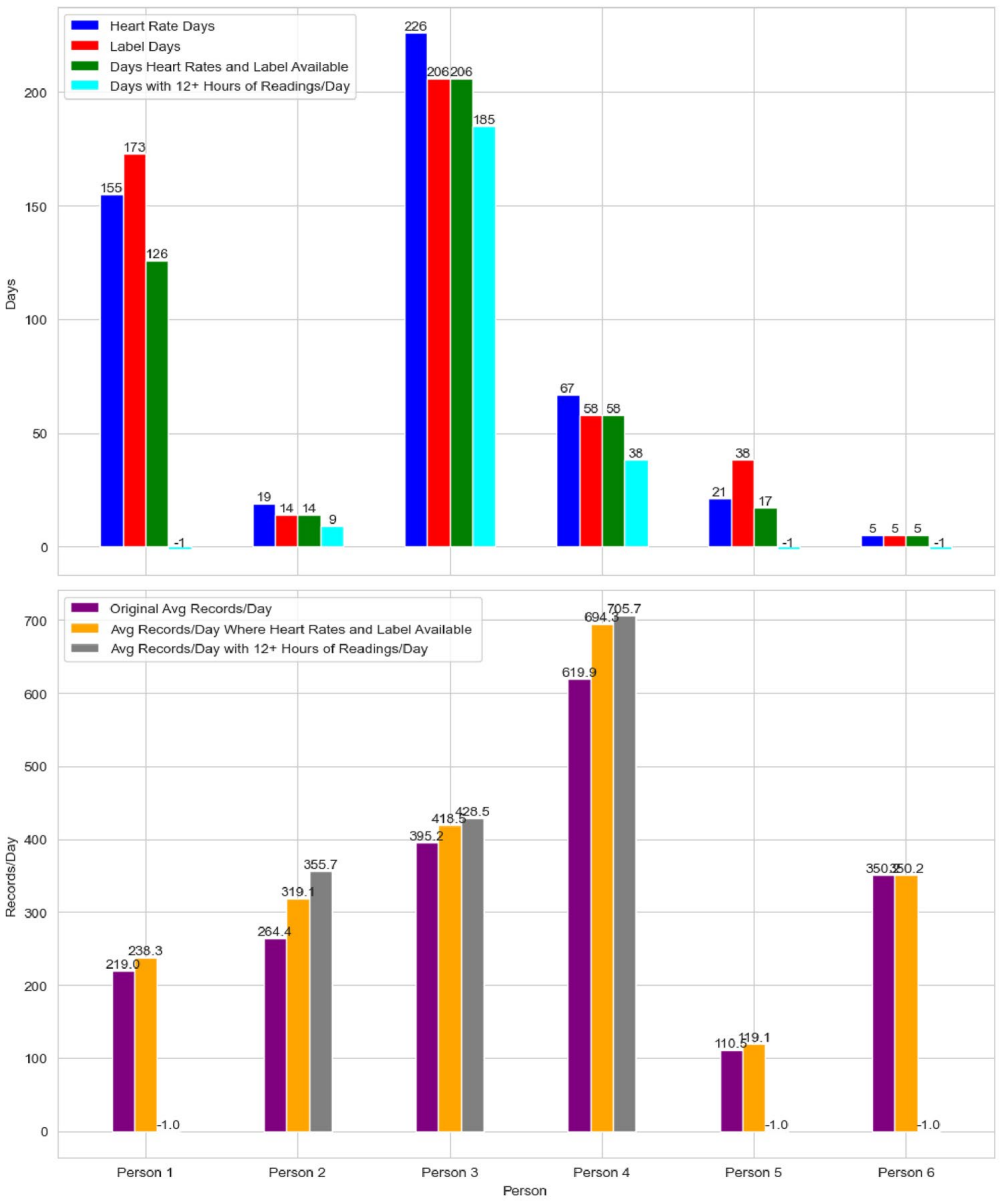
Distribution of record numbers

In Fig. 1, the blue and red columns at the top represent the total number of days with heart rate readings and days with only labels, respectively, for the six participants. Here a “label” refers to a classification or tag indicating the participant’s mental state on a given day, which are collected separately through the in-house built IOS app and provide the ground truth for whether a participant have had a stressful day. The data reveals several patterns: (1) Participants 2, 3, and 4 have more days with heart rate readings than days with labels, likely due to forgetting to input labels. (2) Participants 1 and 5 show more days with labels than heart rate readings, indicating that while labels were manually entered through the app, heart rate readings were missing, possibly because the participants weren’t wearing their Apple Watch at those times. It’s important to note that heart rate readings are only available when wearing the Apple Watch, even though participants may use iPhone throughout the day. (3) Participants 2, 5, and 6 stopped reporting early in the study, whereas Participants 1, 3, and 4 provided more comprehensive data. Notably, Participant 3 used the app consistently for over 200 days out of the 241-day study period. (4) The purple columns at the bottom of Fig. 1 show the average number of daily heart rate readings, which varies significantly among participants, indicating differences in behavior and activity patterns.

### Data filtering

First, we address the mismatch between days with available heart rate readings and those with provided labels, which limits our analysis to days where both types of data are present. Future research could explore unsupervised learning and generative methods [18] to fill in these gaps. The ’green’ columns in top of Fig. 1 show the number of days when both heart rate readings and labels were available for each participant.

**Fig. 2:**
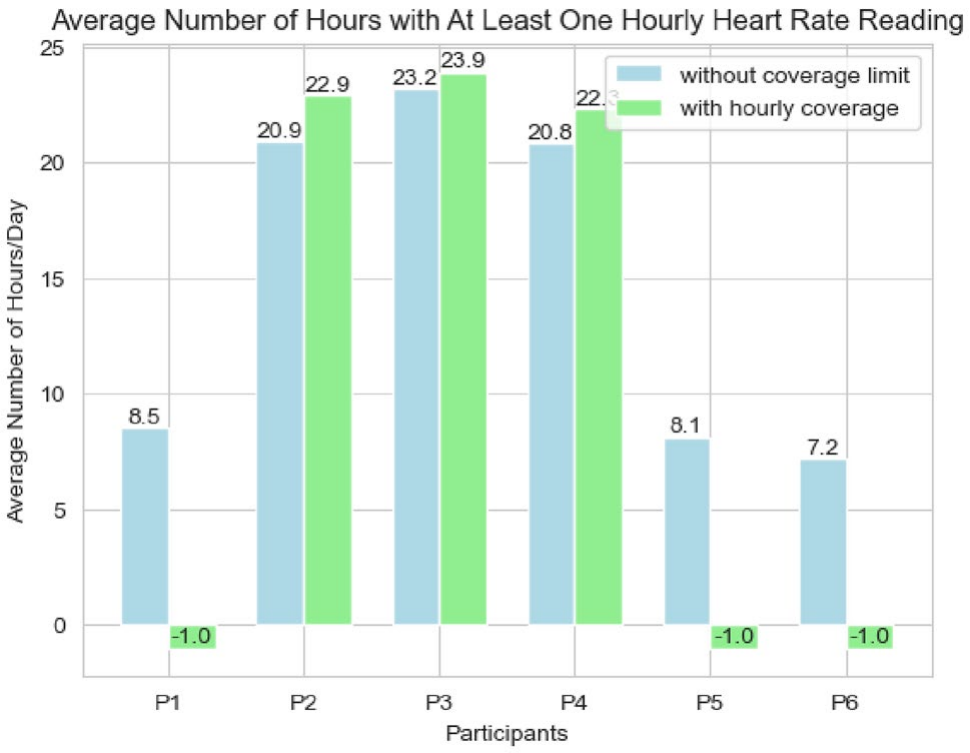
Average number of hours with at least one heart rate reading

Next, we assess whether each day’s heart rate readings provide a comprehensive representation of the participant’s daily behavior. This requires filtering out days where readings are available for only a few hours or limited to specific parts of the day. For instance, if data is recorded only in the morning, afternoon, or for just a few hours, it may not accurately reflect the entire day. Figure 2 shows the average number of hours per day that readings are available, calculated by ensuring at least one reading is present in each hour counted. For Participants 2, 3, and 4, the data is well-distributed, with readings available for around 21 h on average, making their data highly representative of daily activity. Conversely, for Participants 1, 5, and 6, readings are available for fewer than 9 h per day on average, offering a much less complete view of daily behavior.

Requiring heart rate readings for the full 24-h period would be too restrictive. However, to ensure high-quality data and meet the research goal of determining whether heart rate data alone can accurately characterize overall mental states throughout the day, this study includes only days with heart rate readings available for more than 21 out of 24 h. As a result, all records for Participants 1, 5, and 6 are excluded, as indicated by the ’−1’ marker in Fig. 1. Conversely, most records for Participants 2, 3, and 4 are retained. This approach also leads to an increase in the average number of heart rate readings per day, as shown in the bottom chart of Fig. 1.

**Fig. 3:**
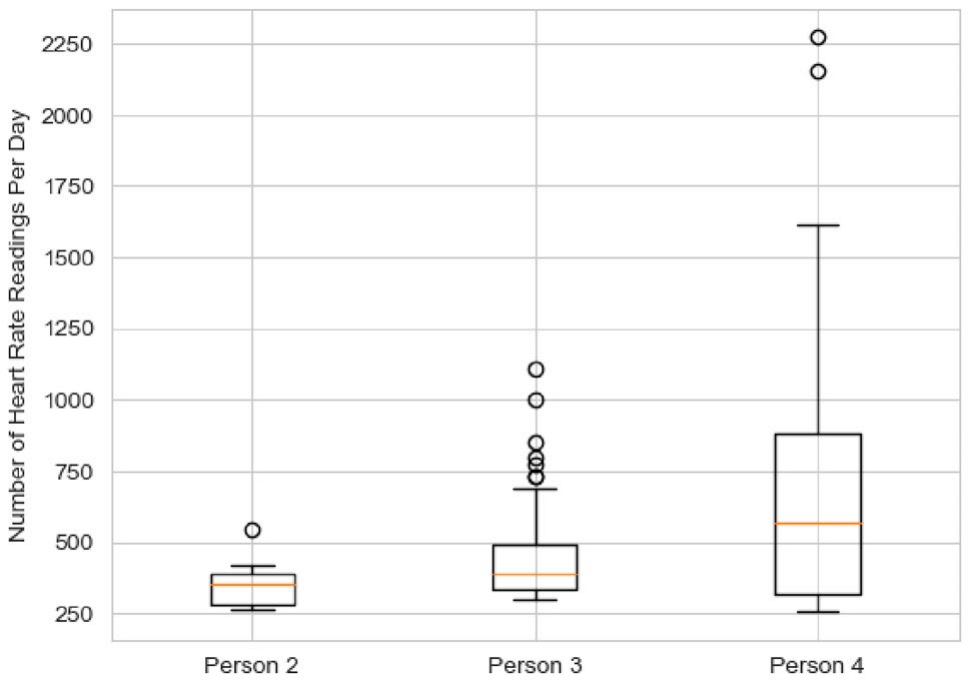
Box plot of number of heart beat readings per day

Finally, we examine the distribution of total heart rate readings available per day using a box plot, as shown in Fig. 3. The number of readings varies significantly among participants, making it difficult to establish a consistent threshold for valid daily readings. On an individual level, Participants 2, 3 and 4 each have some days with an unusually high number of readings, likely due to leaving the exercise mode- which records heart rates more frequently than necessary. However, since these readings are still accurate, and considering the variation in reading patterns across participants, no data has been filtered out at this stage, and all readings are included in further analysis.

In summary, the filtering process identifies valid days for further analysis based on two criteria: (1) both heart rate readings and labels must be available, and (2) heart rate data must be recorded for at least 21 out of 24 h. After applying these filters, 232 days of data from Participants 2, 3 and 4 are retained for further analysis.

### Data sampling and interpolation

This section outlines the sampling and interpolation process applied to the previously selected valid days of heart rate records. The goal of this procedure is to standardize heart rate data, ensuring completeness, and a reliable representation of the user’s physiological state throughout the day.

The core concept of sampling is to capture heart rate readings at regular intervals throughout the day, ensuring that data recorded at different times and frequencies can be aligned and remain consistent for further processing. This approach also facilitates the effective application of subsequent feature transformation using signal processing. Resampling also helps reduce data dimensionality by eliminating the need to retain every individual reading, as long as enough points are captured to represent the overall heart rate patterns. For instance, sampling at 1-min intervals would generate 1440 readings per day, which may be excessive given the limited number of days available for analysis. To address this, the final preprocessed data is downsampled to a 3-min interval, balancing data efficiency and pattern representation.

On the other hand, interpolation is employed to generate missing heart rate readings, addressing the irregular recording intervals of the Apple Watch. This is due to that the device captures more frequent readings during periods of heart rate fluctuation, such as during exercise, and fewer readings when heart rate is more stable such as sleep. Interpolation ensures a more consistent dataset, filling in gaps where data is unavailable.

**Fig. 4:**
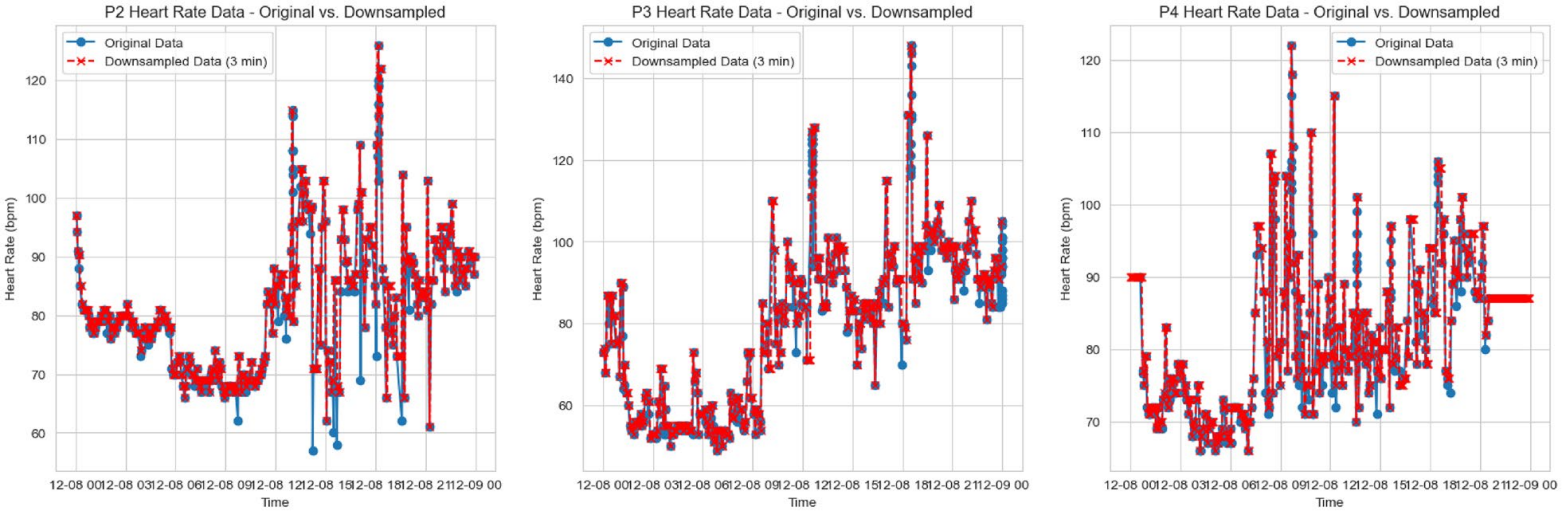
Comparison of original data vs interpolated resamples

The following outlines the data processing steps summarized in the pseudo-code Listing 1, which produce 480 readings per day. Figure 4 presents a comparison between processed data and original data for Participants 2, 3, and 4 on a random day.

1. **Resampling available data**
  Resample heart rate data to ensure that there is exactly one record per minute from the start hour to the end hour.
  In cases where multiple records exist within the same minute, select the maximum value to highlight significant heart rate changes rather than averaging, which might obscure such variations.
2. **Filling missing data**
  - If no record exists for a given minute, fill the gap by taking the immediate last value available. This approach assumes stability in heart rate when no new readings are recorded.
  - For minutes following the last recorded value of the day up to midnight, extend the last recorded value to fill.
  - For minutes before the first recorded value of the day, propagate the first available reading backward to midnight. This typically covers the sleeping hours, assuming minimal heart rate variability.
3. **Downsampling interpolated data**
  - The process involves downsampling previously interpolated 1-min interval data to a user-specified granularity. This reduces data dimensionality, helping prevent overfitting with the limited dataset. Originally, 1-min interpolation and sampling produces 1440 data points per day, so downsampling to 3-min intervals reduces this to 480 readings while preserving key trends for modeling.
  - It’s important to note that directly interpolating at a 3-min interval instead of first at 1-min and then downsampling is not recommended. For example, given heart rate readings [58, ?, 67, ?, ?, ?, ?, 61, 62, ?], direct 3-min interpolation would yield [67, 62], while 1-min interpolation (resulting in [58, 58, 67, 67, 67, 67, 67, 61, 62, 62]), followed by downsampling gives [67, 67]. The latter method is preferred because interpolating at the finer 1-min level more accurately reflects the Apple Watch’s tendency to maintain similar heart rates when there are no significant changes. It also preserves higher readings longer and capturing subtle changes more accurately than broader intervals.

**Listing 1.**
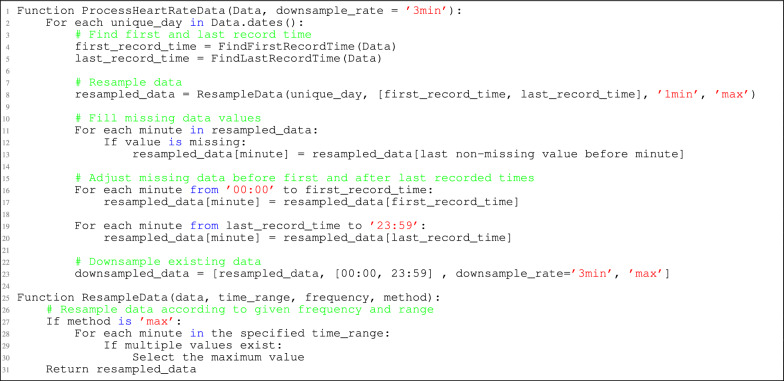
Heart rate data processing

## Methods

Heart rate patterns throughout the day can provide valuable insights into an individual’s daily mental state. Variations in heart rate, such as increases during periods of stress or anxiety, or steady, lower rates during relaxation or sleep, reflect the body’s physiological response to different emotional and mental conditions. Continuous heart rate tracking throughout the day, which may be impacted by different activities and environments, are therefore potentially indicative of daily mental states.

Although the preprocessing steps outlined in Sect. 2 have addressed technical challenges like irregular data collection and missing intervals by using interpolation and resampling to create a consistent time series, significant challenges remain. Even with resampling that reduces the data to 480 readings per day, classifying this long temporal heart rate data is still technically demanding. One challenge lies in managing the high dimensionality and variability of the data, as heart rates can fluctuate widely due to various factors, which complicate the task of identifying consistent patterns that are meaningful for categorizing overall mental states.

It’s worth noting that, in the literature classifying heart disease using heart rate data [19] is generally more straightforward than characterizing mental states, as heart diseases often present with more consistent and predictable patterns. Conditions like arrhythmias or ischemic heart disease tend to produce specific, recognizable alterations in heart rate that can be more reliably identified and categorized [20]. In contrast, using heart rates alone to characterize mental states can be more challenging. The number of different behavioral patterns and the wide range of factors influencing heart rate—such as physical activity, sleep, and environmental conditions—result in a much greater variability in heart rate data. This variability leads to diverse and complex heart rate plots, making it difficult to pinpoint consistent patterns that can be linked to specific mental states. Unlike heart disease, which may manifest through relatively stable indicators, mental states like stress are dynamic and multifaceted, requiring more sophisticated classification models to capture the subtle and often transient changes in heart rate associated with different emotional and psychological conditions.

### Transforming temporal heart rates into frequency domains using Fourier transform

Heart rate sequences alone aren’t typically suitable for direct input into classification models due to their high variability and complex patterns over time. To manage this, extracting features that provide more structured and meaningful information about the data is key. The Discrete Fourier Transform (DFT) [21] is a mathematical technique that converts discrete signals from the time domain to the frequency domain, enabling frequency component analysis. This transformation uncovers frequency characteristics that aren’t readily apparent in time-domain data, where periodic signals and oscillations can be challenging to detect.

When DFT is applied to heart rate data, the time-based sequence of measurements is decomposed into a series of sinusoidal components at different frequencies. This process reveals dominant frequencies and periodic patterns that are otherwise obscured in the time domain. For example, low-frequency components may indicate long-term trends or cycles in heart rate, possibly reflecting baseline physiological states, while higher frequencies may capture short-term variations linked to momentary stressors or physical activity.

The output of the DFT is a set of frequency components, each associated with a specific amplitude and phase. These frequency-based features serve as input for the classification model later, enabling it to leverage information on underlying heart rate patterns rather than just point-by-point variations in time. This approach thus provides a more robust representation of heart rate behavior over time, improving the model’s ability to classify outcomes influenced by temporal heart rate dynamics.

More formally, given a sequence of *N* heart rate measurements $hr_0, hr_1, \ldots , hr_{N-1}$, the Fast Fourier Transform (FFT), which is an efficient algorithm for quickly implementing DFT, which is defined as:

1 $$\begin{aligned} HR_k = \sum _{n=0}^{N-1} hr_n \cdot e^{-i 2\pi k \frac{n}{N}} \end{aligned}$$

where $HR_k$ represents the frequency component at frequency *k* of the heart rate data; $hr_n$ is the heart rate measurement at time point *n*; *N* is the total number of heart rate measurements throughout the day, and *n* varies from 0 to $N-1$, covering all potential frequency components; *e* is the base of the natural logarithm, and *i* is the imaginary unit, with the complex exponentials term $e^{-i 2\pi \frac{n}{N}}$ that oscillate at different frequencies depending on *n*, crucial for separating the heart rate signal into its constituent frequencies. Each $HR_k$ quantifies the strength and phase of the heart rate signal at the frequency *k*. The magnitude $|HR_k|$ reflects the amplitude of that frequency component. To reconstruct the heart rate sequence from its frequency-domain representation, use the Inverse Discrete Fourier Transform:

2 $$\begin{aligned} hr_n = \frac{1}{N} \sum _{k=0}^{N-1} HR_k \cdot e^{i 2\pi k \frac{n}{N}}. \end{aligned}$$

**Fig. 5:**
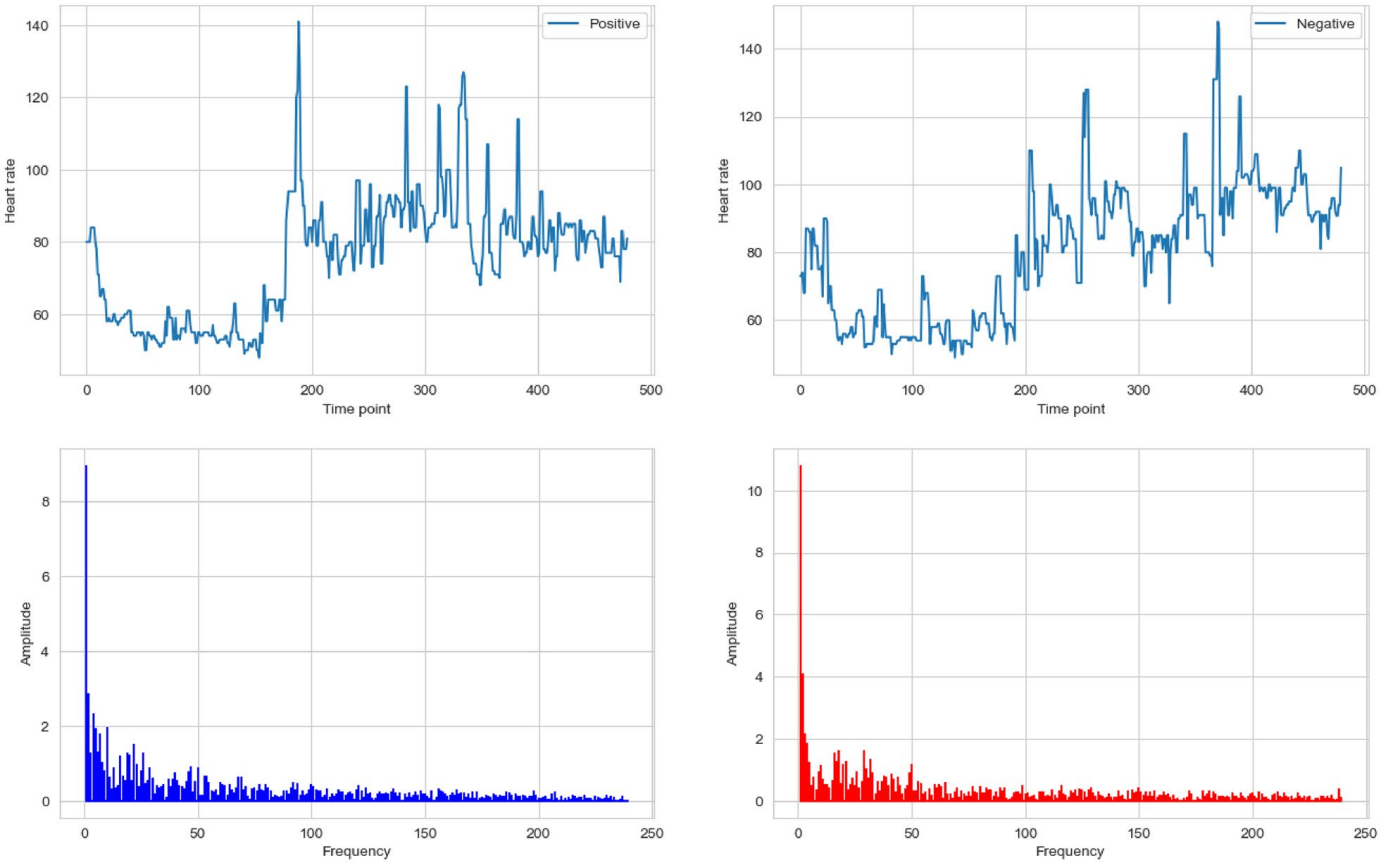
Fourier analysis example

Figure 5 presents two sets of graphs representing heart rate data over 2 random days—one characterized with overall stress (Positive) and the other without overall stress (Negative). Although the Negative day might show somewhat steadier and less volatile with less pronounced peaks, possibly suggesting a calmer physiological state compared to a stressed day, both graphs do show significant fluctuations, with sharp peaks suggesting moments of increased heart rate possibly due to stress-induced situations. In terms of the corresponding Fourier transformed graphs, a slightly higher low-frequency amplitude on a stress day likely indicates a heightened autonomic response, consistent with increased physiological reactivity to stress. The lower amplitude on the non-stress day suggests a more regulated, less reactive state, often associated with rest or baseline physiological function. It’s worth noting that the Fourier transform graphs only show half the frequency spectrum, which is rooted in the properties of the Fourier Transform, when applied to real-valued signals.

### Transforming temporal heart rates into frequency domains using wavelet analysis

Fourier analysis provides an efficient approach to identifying predominant frequency components throughout an entire dataset, offering valuable insights into general patterns. However, it provides only frequency information. In contrast, wavelet analysis enhances this by offering both time and frequency resolution, enabling the detection of transient, non-stationary, or localized features within heart rate data [22]. To complement and expand the analytical capabilities of this research, wavelet analysis is also utilized. Unlike traditional Fourier analysis, wavelet analysis decomposes a signal into components across various scales. This method, particularly through the Discrete Wavelet Transform (DWT), is especially beneficial for heart rate analysis as it captures both the frequency and precise timing of specific events within the signal. This includes sudden heart rate spikes triggered by stress or gradual trends during different phases of physical activity, potentially offering a more nuanced understanding of heart rate dynamics.

Given a heart rate signal $hr[n]$, where $n$ indexes time, the DWT is defined as:

3 $$\begin{aligned} W_{j,k} = \sum _{n=0}^{N-1} hr[n] \cdot \psi _{j,k}[n] \end{aligned}$$

where: $\psi _{j,k}[n]$ is the wavelet function at scale $j$ and position $k$; $j$ controls the dilation, hence affecting the frequency band; $k$ manages the translation, shifting the wavelet across the time series; $N$ is the total number of data points in the heart rate signal.

**Fig. 6:**
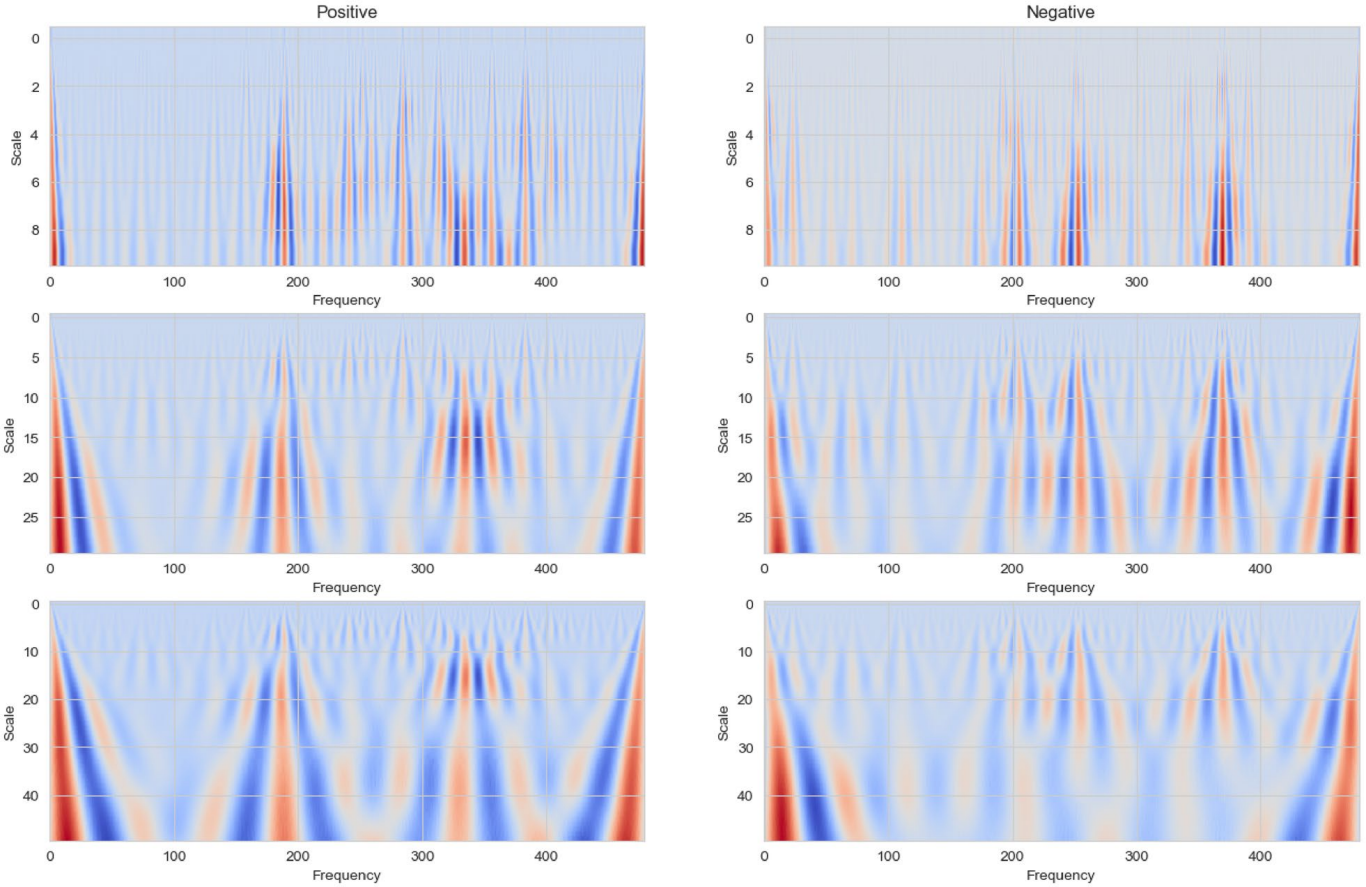
Wavelet analysis example

While a variety of wavelet functions exist, the Morlet wavelet [23] is particularly well-suited for heart rate analysis due to its frequency sensitivity and excellent temporal localization. The Morlet wavelet is a complex sinusoid modulated by a Gaussian window, and is defined as:

4 $$\begin{aligned} \psi (t) = \pi ^{-1/4} e^{i\omega _0 t} e^{-t^2/2} \end{aligned}$$

where $\omega _0$ is the central frequency of the wavelet; $t$ represents time. The Gaussian component ensures localization in both time and frequency domains, making it ideal for handling the non-stationary nature of heart rate signals that fluctuate due to various stimuli.

As illustrated in Fig. 6, the wavelet analysis of heart rate data, captures the possible differences between the previously randomly selected stressful day (Positive) and a non-stressful day (Negative) using three different scales: 10, 30, and 50. On the stress day, the analysis reveals increased heart rate variability and more pronounced fluctuations at all examined scales, indicative of the physiological impact of stress. Conversely, the non-stress day exhibits a more uniform and steady pattern, characterized by reduced variability and fewer intense peaks. The choice of scales—10, 30, and 50—proves effective in highlighting these differences. The smaller scale captures fine details and rapid changes, while larger scales show how these patterns aggregate over more extended periods. It has been noted that the differentiation in patterns between stress and non-stress days, as revealed by wavelet analysis, is more pronounced than what was observed in the earlier FFT analysis. However, a more detailed comparative analysis will be carried out in subsequent experiments to thoroughly assess these observations.

### Mental state modelling with machine learning

Given the original feature vector $x_{\text {org}} \in \mathbb {R}^{480}$ as preprocessed in Sect. 2, the application of the Fast Fourier Transform (FFT) also produced a 480-dimensional vector. However, due to the symmetry that occurs when FFT is applied to real-valued signals, the feature values generated are symmetric about the center. Consequently, only half of these frequencies are necessary to capture the entire spectrum information, effectively reducing the vector size to a 240-dimensional representation.

In the case of wavelet transformations using the Morlet wavelet at different scales, namely 10, 30, and 50, the resulting dimensionalities are: $x_{{\text {wave}}\_10} \in \mathbb {R}^{10\times {480}}$, $x_{ {\text {wave}}\_30 } \in \mathbb {R}^{30\times {480}}$, $x_{ {\text {wave}}\_50} \in \mathbb {R}^{50\times {480}}$. These scales lead to feature spaces that are significantly larger than the original feature vector, posing potential overfitting risks. To address the high dimensionality, Principal Component Analysis (PCA) is applied to each day’s wavelet features $x^i_{\text {wave}}$. It is observed that retaining only 5 principal components maintains 95% (or more) of the original variance across all scales. The reduced dimensionalities are: $x'_{{\text {wave}}\_10} \in \mathbb {R}^{10\times {5}}$, $x'_{{\text {wave}}\_30}\in \mathbb {R}^{30\times {5}}$, $x'_{{\text {wave}}\_50} \in \mathbb {R}^{50\times {5}}$. These vectors can be further flattened, resulting in the extracted features denoted as $x_{\text {wave50}}, x_{\text {wave150}}, x_{\text {wave250}}$.

**Fig. 7:**
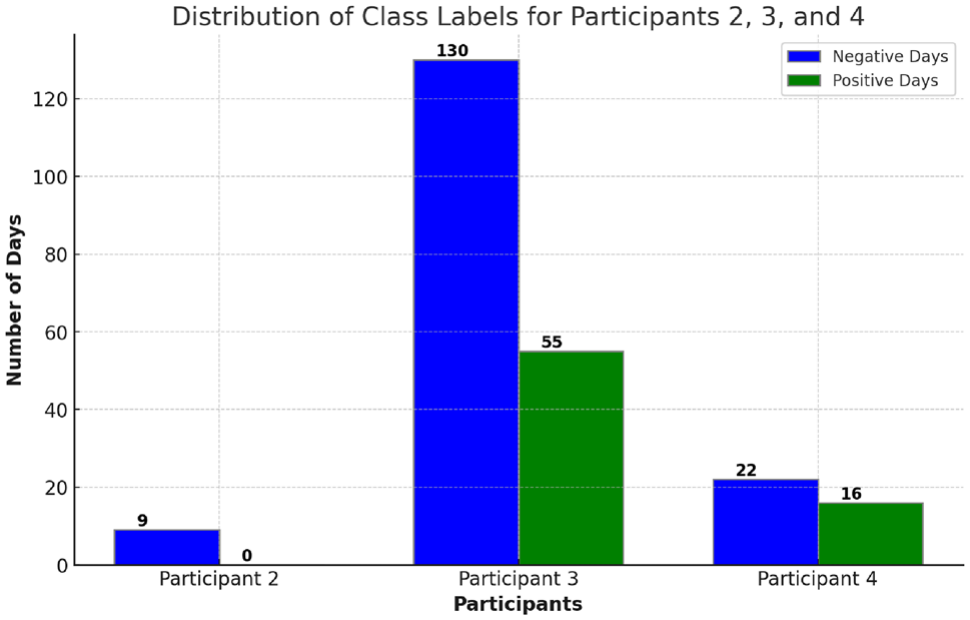
Overall experimental scheme

**Fig. 8:**
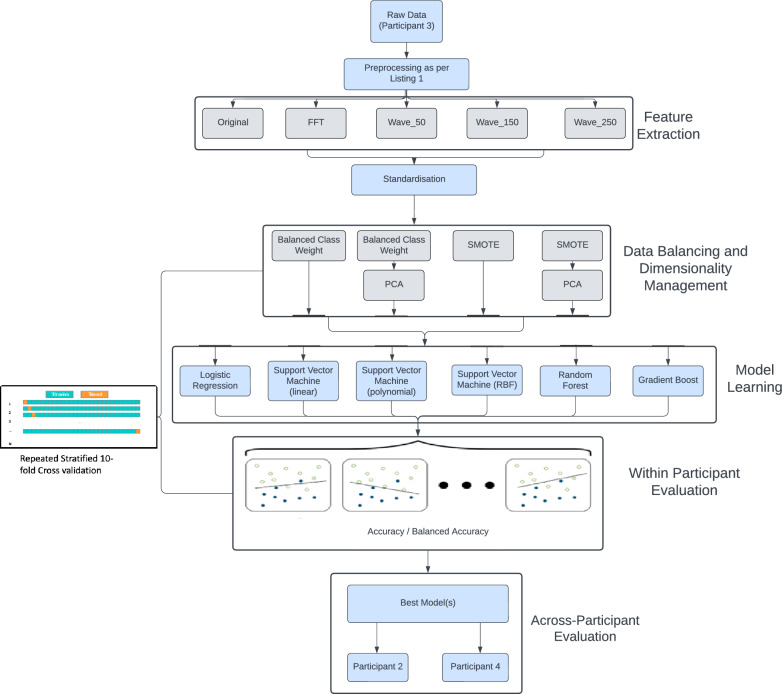
Overall experimental scheme

Machine learning has been proven effective in a wide range of health and care scenarios [24], including studies focused on the effectiveness of wearables in classifying mental states. The primary objective of this research is to evaluate the effectiveness of day-long heart signals in modelling mental states, with a particular focus on detecting stress. For each set of features generated by $x_{\text {org}}$, $x_{\text {fft}}$, $x_{\text {wave50}}$, $x_{\text {wave150}}$, and $x_{\text {wave250}}$, the following experimental settings are implemented in dealing with high-dimensional data as well as imbalanced distribution of stressful vs non-stressful as shown in Fig. 7, ensuring robust and accurate modelling of mental states based on heart rate data.

1. **Setting 1—Classification with balanced class weights (BCW)**: A robust classifier is applied directly to the features with balanced class weights by assigning higher weights to minority classes and lower weights to majority classes during model training. This way, the model penalizes misclassifications of the minority class more heavily, reducing bias towards the majority class while encouraging it to pay more attention to underrepresented examples. This applies to all six classifiers as introduced below.
2. **Setting 2—Classification with balanced class weights and PCA**: Principal Component Analysis (PCA) is employed to reduce the feature dimensionality before classification, which then uses balanced class weights to handle class imbalances for all six classifiers.
3. **Setting 3—Classification with synthetic data generation**: Synthetic Minority Over-sampling Technique (SMOTE) [25] is used to generate synthetic data of the minority class to balance originally uneven class distribution before classification.
4. **Setting 4—Classification with PCA and SMOTE**: SMOTE is used for balancing class distribution, followed by PCA to address dimensionality reduction before classification.

In this study, traditional machine learning classifiers are utilized due to the limited amount of data available, whereas recent advancements in deep learning typically necessitate more extensive datasets to develop reliable models [26]. A range of models is employed to ensure a comprehensive evaluation. Logistic Regression [27] is chosen for its simplicity and general effectiveness in binary classification. The Support Vector Machine (SVM) [28] is implemented as a more powerful classifier, equipped with various kernels to adapt to different data properties: the Linear Kernel for linearly separable data, the Polynomial Kernel to fit non-linear boundaries (adjustable by the polynomial degree), and the Radial Basis Function (RBF) or the Gaussian Kernel, which excels in non-linear scenarios with complex data structures. Random Forest [29] is an ensemble learning model that improves upon single decision trees by building multiple trees and aggregating their predictions. Its inherent randomness in selecting data points and features for each tree ensures diverse model perspectives, contributing to its reliability and generalization capabilities. Gradient Boosting [30] is another ensemble technique that builds models sequentially, with each new model being created to correct the errors made by previous models. Notably, SVM, Random Forest, and Gradient Boosting are often regarded among the most powerful traditional classifiers in machine learning [31, 32].

The evaluation framework utilizes a tenfold cross-validation approach, repeated 10 times randomly in a stratified manner. This repeated stratified cross-validation ensures that each fold of the data represents a good mix of the overall sample, maintaining the original class distribution across each training and testing fold. Specifically, for this study, considering that Participant 3 possesses the most extensive dataset (185 valid days out of 232, nearly 80%), the framework outlined in Fig. 8 is implemented across all 185 records using multiple iterations of 10-fold cross-validation. Moreover, leveraging the optimal setup determined for Participant 3, an additional experiment evaluates how effectively the models, when generalized, perform on data from Participants 2 and 4.

## Results and discussions

**Table 1:** Comparison of classifiers and processing techniques: original vs FFT

Classifier	Preprocessing	Original			FFT		
		Training accuracy	Test accuracy	Balanced test accuracy	Training accuracy	Test accuracy	Balanced test accuracy
		(%) ± Std	(%) ± Std	(%) ± Std	(%) ± Std	(%) ± Std	(%) ± Std
Logistic regression	BCW	100 ± 0	67.2 ± 9.8	60.9 ± 13	**100** ± **0**	**66.8** ± **9.6**	**59.8** ± **13.2**
	BCW+PCA	99.9 ± 0.2	66.8 ± 9.8	59.9 ± 13.3	100 ± 0	66.2 ± 10.3	59.1 ± 14.7
	SMOTE	**100** ± **0**	**67.6** ± **9.9**	**61.2** ± **13.4**	100 ± 0	66.3 ± 8.8	58.8 ± 12.2
	SMOTE+PCA	100 ± 0.1	65.6 ± 10.5	58.1 ± 14.6	100 ± 0	66 ± 9.6	58.9 ± 14.3
SVM (linear)	BCW	**100** ± **0**	**68.2** ± **9.5**	**61.7** ± **13.5**	**100** ± **0**	**67.7** ± **9.4**	**61.2** ± **13.4**
	BCW+PCA	100 ± 0	66.2 ± 10.7	58.6 ± 14.4	100 ± 0	66.8 ± 9.4	59.6 ± 13.3
	SMOTE	**100** ± **0**	**68.2** ± **9.5**	**61.7** ± **13.5**	100 ± 0	65.6 ± 9.7	58.2 ± 15.2
	SMOTE+PCA	100 ± 0	65.5 ± 9.8	57.5 ± 14	100 ± 0	66.8 ± 9.4	59.6 ± 13.3
SVM (rbf)	BCW	96.4 ± 1	69.3 ± 9.7	64.7 ± 11.4	100 ± 0.1	70.5 ± 9.6	65.2 ± 15.8
	BCW+PCA	94.6 ± 0.9	70.3 ± 9.6	65.9 ± 11.8	**99.8** ± **0.3**	**71.8** ± **7.7**	**68.4** ± **17.4**
	SMOTE	99 ± 0.4	71.6 ± 8.4	66.6 ± 15.9	100 ± 0.1	70.2 ± 5	66.4 ± 17.6
	SMOTE+PCA	**98.4** ± **0.6**	**72.5** ± **9.5**	**67.2** ± **15.8**	100 ± 0.1	69.2 ± 9.5	62.5 ± 16.3
SVM (poly)	BCW	**96.6** ± **0.8**	**72.9** ± **6.1**	**72.2** ± **18.2**	96 ± 0.5	70.9 ± 2.4	72 ± 5.3
	BCW+PCA	95.8 ± 1	72.3 ± 6	69.4 ± 18.3	95.6 ± 0.5	70.9 ± 2.4	72 ± 5.3
	SMOTE	98.2 ± 1.3	70.9 ± 8.5	65 ± 16.6	99.1 ± 0.9	71.7 ± 4.7	71.2 ± 15.6
	SMOTE+PCA	98.7 ± 1.2	70.5 ± 8.6	64.4 ± 16.1	**98.5** ± **1.2**	**71.8** ± **3.9**	**72.2** ± **13.9**
Random forest	BCW	**100** ± **0**	**72** ± **6.7**	**68.8** ± **17.8**	**100** ± **0**	**70.4** ± **3.5**	**68.4** ± **12.7**
	BCW+PCA	100 ± 0	70.4 ± 2.4	70.1 ± 7.5	100 ± 0	70.3 ± 2	70 ± 4.1
	SMOTE	100 ± 0	71.2 ± 9.6	65.5 ± 13.9	100 ± 0	69.5 ± 8.1	60.9 ± 19.5
	SMOTE+PCA	100 ± 0	66.8 ± 9.2	55.3 ± 19.3	100 ± 0	69.7 ± 3.1	66.8 ± 12
Gradient boosting	BCW	**100** ± **0**	**73.4** ± **8.3**	**69.3** ± **14.3**	**100** ± **0**	**69.8 ± 8.5**	**62.4 ± 17.9**
	BCW+PCA	100 ± 0	68.6 ± 7.6	58.8 ± 18.1	100 ± 0	69.2 ± 7	62.5 ± 18.8
	SMOTE	100 ± 0	69.9 ± 9.2	63.5 ± 14.2	100 ± 0	66.7 ± 9.7	60.6 ± 14.6
	SMOTE+PCA	100 ± 0	63.2 ± 10.6	54.1 ± 15.5	100 ± 0	67.2 ± 5.8	55.2 ± 18.6

**Table 2:** Comparison of classifiers and preprocessing techniques: Wave_50, Wave_150, and Wave_250

Classifier	Processing	Wave_50			Wave_150			Wave_250		
		Training accuracy	Test accuracy	Balanced test accuracy	Training accuracy	Test accuracy	Balanced test accuracy	Training accuracy	Test accuracy	Balanced test accuracy
		(%) ± Std	(%) ± Std	(%) ± Std	(%) ± Std	(%) ± Std	(%) ± Std	(%) ± Std	(%) ± Std	(%) ± Std
Logistic regression	BCW	**74.4** ± **1.8**	**64.6** ± **10.8**	**61.1** ± **11.6**	**78.7** ± **1.6**	**63.0** ± **10.7**	**58.8** ± **11.1**	**82.6** ± **1.4**	**67.8** ± **10.5**	**64.1** ± **12.3**
	PCA	63.6 ± 1.9	58.4 ± 11.3	55.6 ± 11.4	67.5 ± 1.9	61.3 ± 10.4	58.6 ± 10.8	67.9 ± 2.6	62.4 ± 11.2	60.0 ± 11.6
	SMOTE	74.5 ± 2.2	64.1 ± 10.5	60.8 ± 10.8	81.6 ± 1.9	62.6 ± 10.4	58.3 ± 11.0	84.4 ± 1.8	66.9 ± 10.1	62.8 ± 12.0
	SMOTE+PCA	63.7 ± 2.4	58.5 ± 11.4	55.9 ± 11.5	69.1 ± 2.3	60.2 ± 10.2	57.4 ± 11.0	67.8 ± 3.8	58.7 ± 11.4	56.7 ± 11.3
SVM (linear)	BCW	**77.4** ± **1.8**	**65.6** ± **9.9**	**62.1** ± **10.8**	**79.7** ± **2.1**	**62.5** ± **10.6**	**59.6** ± **10.7**	**84.1** ± **1.5**	**67.6** ± **9.9**	**64.2** ± **10.8**
	PCA	64.3 ± 1.8	58.3 ± 11.7	55.4 ± 11.6	66.7 ± 2.4	61.3 ± 10.6	59.6 ± 10.6	68.0 ± 3.1	59.6 ± 12.0	57.9 ± 12.2
	SMOTE	76.9 ± 2.4	63.4 ± 10.7	60.0 ± 11.3	83.2 ± 2.0	62.4 ± 11.2	58.8 ± 12.2	86.9 ± 1.4	66.5 ± 10.5	63.2 ± 12.2
	SMOTE+PCA	64.2 ± 2.5	58.0 ± 11.1	55.9 ± 10.7	70.0 ± 2.5	59.6 ± 10.3	58.9 ± 10.3	69.3 ± 3.6	55.5 ± 11.4	55.0 ± 11.0
SVM (rbf)	BCW	**74.2** ± **2.8**	**56.8** ± **10.0**	**53.7** ± **9.9**	82.8 ± 1.5	66.2 ± 9.9	63.5 ± 10.9	83.7 ± 1.5	63.7 ± 9.8	60.8 ± 10.1
	PCA	71.7 ± 2.5	55.4 ± 10.8	52.8 ± 10.2	80.6 ± 1.4	64.5 ± 9.8	62.3 ± 10.0	81.9 ± 1.7	63.2 ± 10.9	60.2 ± 11.2
	SMOTE	79.2 ± 1.8	55.7 ± 9.8	53.6 ± 9.2	**87.5** ± **1.4**	**66.8** ± **9.5**	**62.5** ± **10.9**	**87.7** ± **1.3**	**66.2** ± **10.3**	**62.1** ± **11.2**
	SMOTE+PCA	76.6 ± 1.8	55.0 ± 10.5	54.1 ± 9.8	85.1 ± 1.6	65.5 ± 9.0	61.9 ± 9.8	84.2 ± 1.7	63.5 ± 10.5	59.5 ± 11.1
SVM (poly)	BCW	**83.3** ± **1.2**	**70.5** ± **8.4**	**64.0** ± **16.1**	**85.0** ± **0.9**	**71.8** ± **7.0**	**66.7** ± **15.9**	**84.3** ± **1.1**	**65.7** ± **7.8**	**54.0** ± **16.4**
	PCA	81.4 ± 1.4	67.9 ± 8.4	60.6 ± 15.4	83.4 ± 0.9	71.7 ± 7.8	67.0 ± 16.5	83.1 ± 1.2	65.5 ± 8.2	54.7 ± 15.3
	SMOTE	74.8 ± 1.3	69.6 ± 8.6	61.6 ± 15.9	80.6 ± 2.6	69.9 ± 9.2	64.0 ± 14.9	83.3 ± 3.7	63.0 ± 10.7	54.6 ± 14.3
	SMOTE+PCA	72.8 ± 1.5	68.2 ± 9.1	60.9 ± 15.9	80.0 ± 2.6	68.4 ± 9.9	63.6 ± 13.9	81.6 ± 3.1	58.8 ± 11.7	52.1 ± 12.9
Random forest	BCW	**100.0** ± **0.0**	**70.0** ± **7.0**	**61.9** ± **17.8**	100.0 ± 0.0	69.3 ± 6.0	61.9 ± 18.6	100.0 ± 0.0	69.9 ± 5.5	63.0 ± 18.4
	PCA	100.0 ± 0.0	68.9 ± 7.7	60.1 ± 18.9	100.0 ± 0.0	69.0 ± 6.5	61.4 ± 20.1	**100.0** ± **0.1**	**70.0** ± **5.8**	**62.8** ± **18.4**
	SMOTE	100.0 ± 0.0	69.4 ± 9.4	64.1 ± 12.7	**100.0** ± **0.0**	**71.2** ± **9.7**	**65.9** ± **14.3**	100.0 ± 0.0	65.8 ± 8.9	58.6 ± 12.4
	SMOTE+PCA	100.0 ± 0.0	63.2 ± 10.7	56.1 ± 13.7	100.0 ± 0.0	65.2 ± 8.9	57.7 ± 12.8	100.0 ± 0.0	64.3 ± 8.7	57.3 ± 12.6
Gradient boosting	BCW	**100.0** ± **0.0**	**68.0** ± **9.3**	**61.3** ± **13.3**	100.0 ± 0.0	68.2 ± 9.1	61.2 ± 14.4	**100.0** ± **0.0**	**67.1** ± **10.4**	**60.2** ± **15.9**
	PCA	100.0 ± 0.1	61.8 ± 9.9	53.9 ± 13.2	100.0 ± 0.0	65.7 ± 10.0	59.1 ± 13.8	100.0 ± 0.0	65.3 ± 9.5	57.9 ± 13.0
	SMOTE	100.0 ± 0.0	66.1 ± 9.6	60.3 ± 11.7	**100.0 ± 0.0**	**68.4 ± 10.4**	**63.0 ± 13.2**	100.0 ± 0.0	65.3 ± 9.4	58.0 ± 13.7
	SMOTE+PCA	99.9 ± 0.2	61.2 ± 10.6	55.3 ± 13.2	100.0 ± 0.1	64.0 ± 9.4	57.9 ± 11.1	100.0 ± 0.1	63.5 ± 10.1	57.7 ± 12.6

In terms of the within-evaluation for Participant 3, Tables 1 and 2 present the detailed results of various machine learning classifiers across different processing techniques, applied to data modalities including the original data, data processed by Fast Fourier Transform (FFT), and data extracted via Morlet wavelet at three scales: Wave_50, Wave_150, and Wave_250. The classifiers were evaluated under four distinct settings: using balanced class weight (BCW), BCW with Principal Component Analysis (PCA), SMOTE only, and a combination of PCA and SMOTE, as detailed in last section. The performance metrics include training accuracy, testing accuracy, and balanced testing accuracy and associated standard deviation, with a baseline of 70.27% representing the default class distribution between stress and non-stress. Each cell in Tables 1 and 2 is based on 10 repeated runs of random tenfold cross-validation. Additionally, the best testing results for each classifier and data modality are highlighted (for example, using logistic regression with original data features, the highest test accuracy achieved is 67.6%).

**Table 3:** Performance comparison across different preprocessing techniques with averages

		BCW	BCW+PCA	SMOTE	SMOTE+PCA	Averaged
Original	Training accuracy	98.83 ± 0.3	98.38 ± 0.35	99.53 ± 0.28	99.52 ± 0.32	99.07 ± 0.31
	Test accuracy	70.5 ± 8.35	69.1 ± 7.68	69.9 ± 9.18	67.35 ± 9.7	69.21 ± 8.73
	Balanced test accuracy	66.27 ± 14.7	63.78 ± 13.9	63.92 ± 14.58	59.43 ± 15.88	63.35 ± 14.77
FFT	Training accuracy	99.33 ± 0.1	99.23 ± 0.13	99.85 ± 0.17	99.75 ± 0.22	99.54 ± 0.15
	Test accuracy	68.87 ± 7.22	69.45 ± 6.35	68.32 ± 7.92	68.5 ± 6.75	68.78 ± 7.06
	Balanced test accuracy	64.18 ± 13.53	65.65 ± 12.03	62.73 ± 16.2	62.52 ± 14.38	63.77 ± 14.04
Wave_50	Training accuracy	84.88 ± 1.27	80.17 ± 1.28	84.23 ± 1.28	79.53 ± 1.4	82.20 ± 1.31
	Test accuracy	65.92 ± 9.23	61.78 ± 9.97	64.72 ± 9.77	60.68 ± 10.57	63.28 ± 9.88
	Balanced test accuracy	60.68 ± 13.25	56.4 ± 13.45	60.07 ± 11.93	56.37 ± 12.47	58.38 ± 12.78
Wave_150	Training accuracy	87.7 ± 1.02	83.03 ± 1.1	88.82 ± 1.32	84.03 ± 1.52	85.90 ± 1.24
	Test accuracy	66.83 ± 8.88	65.58 ± 9.18	66.88 ± 10.07	63.82 ± 9.62	65.78 ± 9.44
	Balanced test accuracy	61.95 ± 13.6	61.33 ± 13.63	62.08 ± 12.75	59.57 ± 11.48	61.23 ± 12.87
Wave_250	Training accuracy	89.12 ± 0.92	83.48 ± 1.45	90.38 ± 1.37	83.82 ± 2.05	86.70 ± 1.45
	Test accuracy	66.97 ± 8.98	64.33 ± 9.6	65.62 ± 9.98	60.72 ± 10.63	64.41 ± 9.8
	Balanced test accuracy	61.05 ± 13.98	58.92 ± 13.62	59.88 ± 12.63	56.38 ± 11.92	59.06 ± 13.04
Averaged	Training accuracy	91.97 ± 0.72	88.86 ± 0.86	92.56 ± 0.88	89.33 ± 1.1	Baseline =
	Test accuracy	67.82 ± 8.53	66.05 ± 8.56	67.09 ± 9.38	64.21 ± 9.45	70.27
	Balanced test accuracy	62.83 ± 13.81	61.22 ± 13.33	61.74 ± 13.62	58.85 ± 13.23	

To ease the interpretation of the extensive information presented in Tables 1 and 2, a summary Table 3 is also provided, which averages the results across classifiers, aiming to facilitate the identification of the most effective data processing techniques and the data modalities that yield the best performance. From the holistic level, the following high-level observations can be made:

- Training accuracies are consistently high, especially when using the original data or data processed by FFT, often reaching nearly 100%. However, when training on data extracted via wavelet analysis, accuracies are generally lower, around 85%. Considering the baseline of 70.27%, this indicates that these models are effectively learning from the training data. However, the near-perfect accuracies with the original and FFT-processed data may suggest a higher likelihood of severe overfitting, particularly when considered with the lower test performance, as discussed below.
- Testing accuracy consistently falls well below training accuracy across all scenarios, highlighting the challenges in generalizing from the training set to unseen data. FFT preprocessing results in slightly lower testing accuracy (68.78%) compared to the original data (69.21%), both of which are below the baseline of 70.27%. Wavelet-transformed data (Wave_50, Wave_150, Wave_250) performs even worse, with testing accuracies around 65%. Note the fact that the averaged accuracies across various data modalities below the baseline do not necessarily imply that FFT and wavelet transformations are ineffective in this context. Instead, it suggests that the complexity introduced by these methods might not be universally beneficial when applied broadly. It’s important to note that the current analysis averages results across different settings and features, which may dilute the potential effectiveness of FFT and wavelet methods. These transformations could perform better in specific scenarios or when used in combination with particular settings, as can be seen in Tables 1 and 2. Between test accuracy and balanced test accuracy, the latter one, which is the average of the recall (or true positive rate) for each class, is consistently lower than the test accuracy, suggesting machine learning may put more emphasis on predicting majority classes.
- In terms of the general best setting for class imbalance and dimensionality reduction, When addressing class imbalance, the use of balanced class weights appears slightly more effective than SMOTE, as indicated by the averaged results. This may be because the Euclidean distance used by SMOTE does not accurately capture the true similarity between points in the high-dimensional data used in this study, leading to the creation of synthetic samples that may not be meaningful or useful. Class balance weighting may be preferred, as it adjusts the classifier’s loss function without altering the data, so may be suited for high-dimensional data without relying on generating new samples. Regarding dimensionality reduction, incorporating PCA alongside either approach to handling imbalanced classes actually leads to decreased performance, suggesting that PCA does not improve generalization capability in this high-dimensional data context.

In summary, the optimal approach on average is to address class imbalance, with balanced class weighting slightly outperforming SMOTE. The use of PCA does not enhance discriminative capability. Regarding data modalities, the original features deliver the best average performance, followed closely by FFT with a slight decrease, while Morlet wavelet transformations across the three scales perform worst. Examining more closely based on more detailed settings, as per Tables 1 and 2, the following observations are drawn:

- When using the original data, the best performances are achieved with Random Forest, Gradient Boosting, and Support Vector Machine (using polynomial and RBF kernels), all of which reach or exceed 72%. In contrast, Logistic Regression and SVM with a linear kernel achieve around 68%. This trend is consistent across other data modalities, where more powerful classifiers such as Gradient Boosting, Random Forests, and SVMs with non-linear kernels consistently deliver the best results.
- The highest test accuracy with the original data is 73.4%, achieved using Gradient Boosting, while the top balanced test accuracy is 72.2%, obtained through SVM with a polynomial kernel. For FFT-transformed data, the best test accuracy is 71.8%, also from SVM with a polynomial kernel, which similarly delivers the highest balanced accuracy at 72.2%. For Wave_50, the highest test accuracy is 70.5%, again with SVM (poly kernel), though the balanced accuracy drops to 64%. With Wave_150, the top test accuracy of 71.8% is also achieved by SVM with a polynomial kernel. Notably, both Wave_50 and Wave_150 maintain training accuracies close to or equal to 85%. However, for Wave_250, SVM with a polynomial kernel achieves a similar training accuracy around 85%, but the testing performance drops significantly to 65.7%, indicating overfitting. The best test accuracy with Wave_250 is 70%, achieved by Random Forest, further suggesting that the Wave_250 modality leads to overfitting, resulting in poorer generalization capability.
- Overall, SVM with polynomial kernels consistently delivers the best performance across various data modalities. Specifically, when applied to wavelet-transformed data, SVMs achieve the top results across different settings while maintaining lower training accuracies (generally around 85%) compared to the nearly 100% training accuracies observed with ensemble methods that yield similar results. However, when SVMs are used with original or FFT-transformed signals, they still exhibit significant overfitting. Despite slightly lower performance with wavelet transformations, the fact that SVM with polynomial kernels maintains much lower training accuracies compared to ensemble methods suggests that SVM with a polynomial kernel could be a strong first choice for modeling heart rate data. However, wavelet transformations that generate an excessive number of features, such as Wave_250—which is the only data modality that does not exceed the baseline of 70.2%—should be avoided due to severe overfitting, which significantly undermines the model’s generalization ability.

**Fig. 9:**
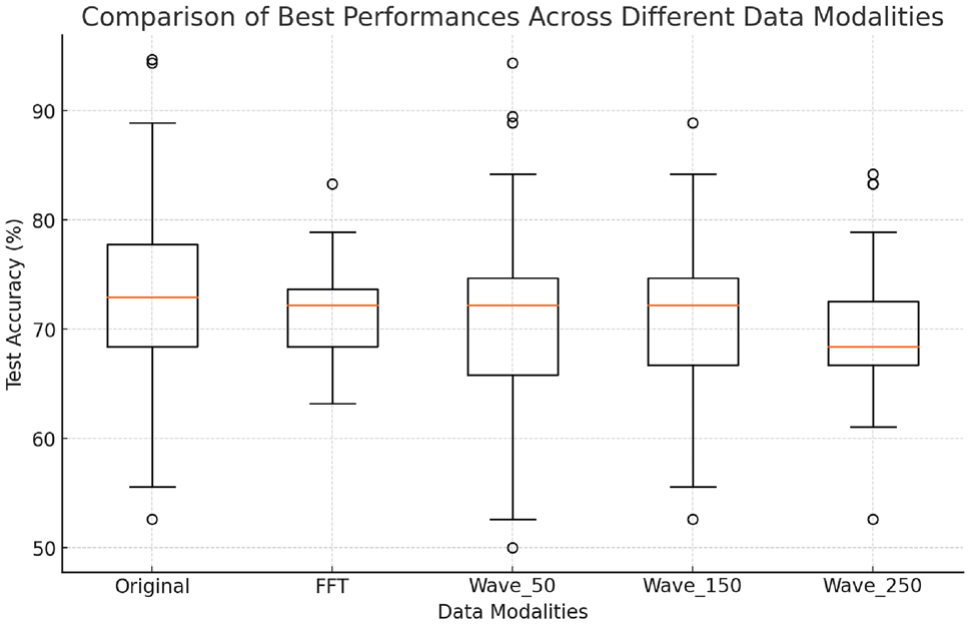
Box plot of best performance across data modalities

### Statistical significance analysis

While the detailed averaged performances have been summarised in Tables 1 and 2, to effectively compare the top performances across different machine learning models, a box plot is utilized presented in Fig. 9, displaying the test accuracy from the top-performing setups for each data modality. Specifically: **Original** data modality’s best results stem from using gradient boosting with balanced class weight. **FFT** achieves its best performance through the use of SVM with a polynomial kernel under PCA and SMOTE. **Wave_50** and **Wave_150** both reach their optimal outcomes by employing SVM with a polynomial kernel under balanced class weight. **Wave_250** records its highest accuracy with Random Forest, coupled with balanced class weight and PCA. Based on 10 random runs of tenfold cross-validation, each modality’s best performance is derived from these 100 observations.

Overall, the box plot visually represents the distribution of results across 100 observations. The **Wave_250** modality shows the poorest performance, while the other four data modalities are relatively similar. However, to determine if different machine learning methods applied to a dataset showed statistically significant differences in test accuracies, a Kruskal-Wallis test [33] was conducted. This non-parametric test, chosen for its suitability in comparing multiple groups without assuming a normal distribution, yielded a statistic of 13.752 and a p-value of 0.0081. The result, indicating significant differences among the group medians, suggested that at least one method performed significantly differently from the others. This prompted further investigation through post-hoc pairwise comparisons using Dunn’s test with Bonferroni correction to identify specifically which methods differed significantly [34]. The combination of these tests provided a comprehensive statistical assessment, initially confirming overall differences with the Kruskal-Wallis test and then pinpointing the specific groups between which these differences occurred through Dunn’s pairwise tests with corrected p-values.

**Table 4:** Comparison of data modalities based on test statistics and significance

	Original	FFT	Wave_50	Wave_150	Wave_250
Original	–	1.64 (No)	0.17 (No)	2.02 (No)	0.02 (Yes)
FFT	–	–	1.37 (No)	6.76 (No)	0.09 (No)
Wave_50	–	–	–	1.68 (No)	7.11 (No)
Wave_150	–	–	–	–	0.25 (No)
Wave_250	–	–	–	–	–

Table 4 presents the pair-wise comparison of best performance achieved by each data modalities, where each cell indicates the adjusted p-value from the Dunn’s test with Bonferroni correction and whether the result shows a significant difference between the methods compared. It can be observed that only the comparison between the “Original” and the “Wave_250” showed a statistically significant difference, with an adjusted p-value of 0.02. This significant result indicates that the performance the original heart rate series is statistically better than that extracted by Morlet wavelet with 50 scales. Conversely, all other pairwise comparisons among the other data modalities did not reveal any significant differences, as their adjusted p-values exceeded the 0.05 threshold, even after applying the stringent Bonferroni correction. This suggests that, apart from the one notable exception, the test accuracies of the different data modalities are statistically similar.

To evaluate whether the best performances of different data modalities significantly outperformed a baseline accuracy of 70.27%, one-sample t-tests [35] was conducted for each data modality. This test was chosen because it’s well-suited for comparing the mean of a sample to a known value, under the assumption that the data are normally distributed, which is a typical condition for accuracy data in machine learning settings. The results as summarised in Table 5 revealed that the accuracies of the “Original” and “FFT” and ’Wave_150’ approaches were statistically significant compared to the baseline, affirming their effectiveness with a high degree of statistical confidence. The ’Wave_50’ and ’Wave_250’ methods do not show statistically significant differences from the baseline. For The Wave_50 and Wave_250, they do not show statistically significant differences from the baseline.

**Table 5:** T-test results on comparative analysis of the top-performing models for each data modality

Method	T-statistic	P-value	Significance
Original	3.83	0.00023	Statistically significant
FFT	3.99	0.00013	Statistically significant
Wave_50	0.22	0.828	Not significant
Wave_150	2.14	0.035	Statistically significant
Wave_250	−0.45	0.651	Not significant

### Evaluating winning data modalities using Participant 2 and 4

The above statistical tests demonstrate that the “Original,” “FFT,” and “Wave_150” methods statistically surpass the baseline performance. However, there is no statistically significant difference in performance among these three methods as confirmed by Table 4. In this subsection, we train a machine learning model using these three top-performing model. The goal is to evaluate their effectiveness in predicting mental states across different participants, specifically Participant 2 and Participant 4, employing the optimal settings that previously yielded the highest performance for each modality from Participant 3.

Table 6 presents the performance of models, over data collected from two other participants, using the three data modalities, each optimized based on previously identified settings. For Participant 2, involving only 9 days all labeled as negative, the models achieve high performance. However, performance significantly declines when applied to Participant 4, who has a more balanced label distribution with 22 negative and 16 positive days, differing notably from Participant 3 used in training. A detailed analysis of the confusion matrix, as shown in last column of Table 6, reveals a strong bias in all three methods towards predicting negative labels. Although the original modality slightly outperforms on independent test sets, its generalization capabilities, given the small test size, remain to be conclusively assessed; while the smallest training-to-test performance gap observed in the wavelet signals aligns with previous findings and indicates a lower likelihood of overfitting that might potentially achieve more robust performance when more data available.

**Table 6:** Accuracy (%) metrics and confusion matrices for different methods

	Training accuracy	Test accuracy on P2	Test accuracy on P4	Confusion matrix on P4
Original	100	100	63.2	$$\begin{bmatrix} 19 & 3 \\ 11 & 5 \end{bmatrix}$$
FFT	100	77.8	57.9	$$\begin{bmatrix} 22 & 0 \\ 16 & 0 \end{bmatrix}$$
Wave	85.4	88.9	57.9	$$\begin{bmatrix} 21 & 1 \\ 15 & 1 \end{bmatrix}$$

## Limitations and future research

This study, utilizing heart rate data from students at a public university in the UK, explored the viability of using heart rates alone to predict day-long stress, employing Fourier transform, wavelet analysis, and machine learning techniques. Experimental results revealed that models using the original heart rates, as well as those transformed by FFT and the Morlet wavelet with 30 scales, yielded statistically significant outcomes compared to the baseline, affirming the effectiveness of signal processing and machine learning in analyzing extended heart rate sequences. Further evaluations on two independent participants show potentials, though there was a noticeable bias towards negative labels. Notably, the models based on wavelet signals exhibited the smallest discrepancy between training and test performances. This consistency for both across and within participant evaluations suggests a lower risk of overfitting when using wavelet analysis and points to the potential for more reliable results as more data becomes available. While this research establishes an understanding of how signal processing and machine learning may be leveraged to interpret heart rate data for mental state assessment, it also outlines the limitations and directions for future work.

A primary limitation of this study is the limited availability of data, particularly the scarcity of valid daily records, which heightens the risk of model overfitting. A significant number of days are deemed invalid due to incomplete heart rate data, often missing several hours of recordings—a prevalent issue in this field. For example, Participant 1’s entire 172 days of records were considered invalid, mostly because less than 21 h of heart rate data were captured daily. This challenge highlights the necessity of employing advanced interpolation techniques or generative approaches to fill in or augment missing data, thus increasing the number of days that may be used for model training. The analysis predominantly focuses on Participant 3, who provides the most comprehensive dataset, facilitating an in-depth examination. However, this may limit the generalizability of the findings to other participants with less data. Efforts to test the models on additional participants are beneficial but the small sample size still constrains the wider applicability of the results. Future studies should aim to include more diverse datasets to enhance the robustness and relevance of the findings across different populations.

Additionally, the study’s methodology of processing day-long heart rate signals through FFT and wavelet analysis on a day-by-day basis fails to consider the potential temporal relationships between consecutive days. Such an approach neglects how the mental state from one day could impact the following day. Future research should explore strategies that incorporate these temporal dependencies to provide a more comprehensive understanding of daily mental state fluctuations.

Another consideration is the study’s sole reliance on heart rate data. While this focus was intentional, as heart rate variability has been extensively linked to stress and emotional states, incorporating additional health-related metrics, such as physical activity levels, sleep quality, and environmental factors, could further enhance the model’s ability to differentiate between mental states. Including such complementary data in future work could create a more robust and nuanced model, improving its accuracy in capturing the complex interplay of factors that contribute to mental states. Despite this, the method of flattening temporal features into frequency domain representations is particularly valuable. It simplifies the integration of heart rate data with other non-temporal health or environmental factors, which might be recorded less frequently, paving the way for a more holistic and effective model for assessing daily mental states.

## Data Availability

Data cannot be shared publicly because of confidential nature of university student information.
